# A Systematic View of the *MLO* Family in Rice Suggests Their Novel Roles in Morphological Development, Diurnal Responses, the Light-Signaling Pathway, and Various Stress Responses

**DOI:** 10.3389/fpls.2016.01413

**Published:** 2016-09-27

**Authors:** Van N. T. Nguyen, Kieu T. X. Vo, Hyon Park, Jong-Seong Jeon, Ki-Hong Jung

**Affiliations:** ^1^Graduate School of Biotechnology and Crop Biotech Institute, Kyung Hee UniversityYongin, South Korea; ^2^Exercise Nutrition and Biochem Lab, Kyung Hee UniversityYongin, South Korea

**Keywords:** abiotic stress, meta-expression analysis, MLO, rice, phylogenomic analysis

## Abstract

The Mildew resistance Locus O (MLO) family is unique to plants, containing genes that were initially identified as a susceptibility factor to powdery mildew pathogens. However, little is known about the roles and functional diversity of this family in rice, a model crop plant. The rice genome has 12 potential MLO family members. To achieve systematic functional assignments, we performed a phylogenomic analysis by integrating meta-expression data obtained from public sources of microarray data and real-time expression data into a phylogenic tree. Subsequently, we identified 12 *MLO* genes with various tissue-preferred patterns, including leaf, root, pollen, and ubiquitous expression. This suggested their functional diversity for morphological agronomic traits. We also used these integrated transcriptome data within a phylogenetic context to estimate the functional redundancy or specificity among OsMLO family members. Here, *OsMLO12* showed preferential expression in mature pollen; *OsMLO4*, in the root tips; Os*MLO10*, throughout the roots except at the tips; and *OsMLO8*, expression preferential to the leaves and trinucleate pollen. Of particular interest to us was the diurnal expression of *OsMLO1, OsMLO3*, and *OsMLO8*, which indicated that they are potentially significant in responses to environmental changes. In *osdxr* mutants that show defects in the light response, *OsMLO1, OsMLO3, OsMLO8*, and *four* calmodulin genes were down-regulated. This finding provides insight into the novel functions of MLO proteins associated with the light-responsive methylerythritol 4-phosphate pathway. In addition, abiotic stress meta-expression data and real-time expression analysis implied that four and five *MLO* genes in rice are associated with responses to heat and cold stress, respectively. Upregulation of *OsMLO3* by *Magnaporthe oryzae* infection further suggested that this gene participates in the response to pathogens. Our analysis has produced fundamental information that will enhance future studies of the diverse developmental or physiological phenomena mediated by the MLO family in this model plant system.

## Introduction

The heritable significance of the Mildew resistance Locus O (MLO) family of seven-transmembrane proteins was first recognized in barley (*Hordeum vulgare*), where its loss of function caused resistance response against the powdery mildew pathogen *Blumeria graminis* f. sp. *hordei* (*Bgh*) (Jorgensen, 1992; Büschges et al., 1997). The MLO family is believed to be unique to plants and green algae (Devoto et al., 1999; Kim et al., 2002b). *MLO* gene family members in 22 land plant species including nine dicots and six monocots have been identified and the phylogenetic classification was performed (Kusch et al., 2016).

The *MLO* genes were originally found in the monocot barley. Homozygous mutant alleles of *HvMLO* are resistant to well-known fungal powdery mildew pathogens in that crop (Jorgensen, 1992; Piffanelli et al., 2002). Its expression can be regulated by many biotic and abiotic effectors, including *Bgh* fungus, leaf wounding, and the herbicide paraquat (Piffanelli et al., 2002). However, functional characterization of this family in dicots has been relatively limited. In *Arabidopsis, AtMLO2* (one of 15 *AtMLO* members), has a role in conferring partial resistance to the powdery mildew pathogens *Golovinomyces orontii* and *G. cichoracearum*. Furthermore, a triple mutant of three paralogs—*AtMLO2, AtMLO6*, and *AtMLO12*—shows complete resistance to a fungal pathogen whereas single mutants of either *atmlo6* or *atmlo12* and the *atmlo6/atmlo12* double mutant exhibit a phenotype with a percentage of host cell entry that is almost the same as that of wild-type (WT) plants (Consonni et al., 2006). This finding indicates that an unequal genetic redundancy might exist with respect to the modulation of defense between *AtMLO* paralogous genes. Therefore, further analyses remain to identify the most predominant member among those members which might facilitate the functional characterization through loss of function approaches.

In addition to the roles that MLO have in defenses against powdery mildew disease, they might also participate in morphological and developmental processes, e.g., root thigmomorphogenesis (Chen et al., 2009; Bidzinski et al., 2014), pollen tube reception in the ovary (Kessler et al., 2010), and pollen hydration (Yi et al., 2014). Shared genetic functions between closely linked family members within the same phylogenetic clade have also been reported but not yet clearly explained. For example, *atmlo4* and *atmlo11* mutant seedlings display a spiral-like root phenotype on minimal and mildly acidic media when they contact a hard surface. However, another close member of the same clade, *AtMLO14*, does not seem to be involved in this phenomenon (Chen et al., 2009). Although it is difficult to gain a complete understanding of the functional complexity within this gene family, it can be estimated by using gene expression patterns integrated into the phylogenetic tree context.

Since the completion of the rice genome annotation project, Liu and Zhu (2008) have identified and isolated 12 *MLO* genes in rice. However, that investigation focused primarily on their evolution, divergence, and structural features, and little was done to obtain clues about their predicted functions. In fact, the phenotype of only one of those 12 *OsMLO*s has been described. Nevertheless, because *MLO* genes in other species seem to be involved in many physiological processes, it is possible that we might be able to discover new phenotypes by evaluating expression profiles in rice as well.

Systematic assignments facilitate the functional identification of individual members within a gene family. Phylogenomic approaches that integrate diverse transcriptome data within the phylogenetic tree context are advantageous when conducting functional studies. For example, we have performed genome-wide phylogenomic analyses of families for heat shock transcription factors, heat shock protein 70, ABC transporters, *crRLK1L* kinase, and aquaporins in rice, and our results have suggested that individual members in each family showed functional diversity or redundancy, based on the integrated intensive transcriptome data to the context of phylogenetic tree (Jin et al., 2013; Nguyen et al., 2013; Nguyen Q. N. et al., 2014; Nguyen V. N. T. et al., 2014).

Like those earlier studies, we conducted such an analysis of the rice MLO family along with a comparative phylogenetic analysis. This included 12 rice MLOs, 15 *Arabidopsis* MLOs, and two barley MLOs. Data related to anatomical meta-expression, as well as responses to abiotic and biotic stresses, were examined within the context of a phylogenetic tree. Our objective was to estimate the functional redundancy or dominance among MLO family members and to gain extensive functional information about those genes. Another of our earlier investigations focused on light-responsive genes (Jung et al., 2008), in which we analyzed the expression patterns and defective phenotypes of light responsive family genes under various light conditions. We demonstrated there the benefit of selecting the predominant gene because defective phenotypes are revealed in loss-of-function mutants. Until now, function of a *MLO* gene (*OsMLO12*) in rice was only reported associated with pollen germination process (Yi et al., 2014). Based on the extensive expression data, we now propose that rice MLO members play broad roles in response to diverse environmental challenges, including pathogen infection, and cold and heat stresses, as well as the light response and developmental processes associated with the root, leaf, and pollen. Detailed data analysis and discussion will be presented.

## Materials and methods

### Multiple alignment and phylogenetic analysis

To perform our phylogenetic analysis of the MLO family, we used the protein sequences of 12 *MLO* genes identified in a previous global analysis of the rice MLO family (Liu and Zhu, 2008). We also developed a tree with rice, barley, and *Arabidopsis* MLO proteins, based on previous global analyses of this gene family (Jorgensen, 1992; Devoto et al., 2003). The protein sequences for our phylogenomic analysis were downloaded from TAIR, GreenPhyl and the Rice Genome Annotation Project Website (Rhee et al., 2003; Ohyanagi et al., 2006; Rouard et al., 2011). After multiple-alignment of those sequences with ClustalX (Higgins et al., 1996), we generated a phylogenetic tree using the Neighbor-Joining method, as incorporated in the MEGA5 tool kit for phylogenetic analysis (Tamura et al., 2011).

### Analysis of microarray data and heatmap development

Microarray data including Affymetric and Agilent array data were downloaded from the NCBI Gene Expression Omnibus (http://www.ncbi.nlm.nih.gov/geo/) and Genevestigator (https://genevestigator.com/gv/) (Zimmermann et al., 2004; Cao et al., 2012). We then uploaded the normalized data to the Multi Experiment Viewer (http://www.tm4.org/mev.html) and visualized the data via heatmaps. We used Genevestigator to compare levels of gene expression in several organs and to estimate the functional similarity among rice, barley, and *Arabidopsis* members.

### Plant materials

Circadian rhythms and functional associations with *rice gigantea* (*OsGI*) were examined using wild-type (WT) plants and *osgi* mutants. Ten-day-old rice seedlings were grown on a Murashige Skoog medium under controlled conditions (28°C/25°C day/night, continuous light, and 78% relative humidity). They were then transferred to individual pots and placed in a growth chamber. Beginning at 30 d after germination, their leaves were sampled at 4-h intervals for 48 h. Light- and dark-dependent expression was analyzed using a heterozygous *osdxr* (rice *1-deoxy-D-xylulose 5-phosphate reductoisomerase*) mutant line for which seeds were germinated as described above. Homozygous and WT plants were identified through genotyping, and leaves were collected from 10-day-old plants. Primers used for genotyping analysis are presented in Table S1.

### Stress treatments and pathogen inoculation

Cold treatment consisted of transferring 10-day-old plants originally grown at 28°C to a 6°C ± 1°C lighted refrigerator. The control plants remained at 28°C. After 48 h, the chilled plants were returned to the 28°C chamber for recovery. Samples were collected at Hours 0, 24, and 48 of low-temperature treatment and after 24 and 48 h of recovery. To simulate heat stress, we exposed 10-day-old plants to 42°C ± 1°C for 0, 3, 6, and 12 h. The control plants remained at 28°C. Leaves of three plants were pooled for one biological replicate and each treatment had three repeats. To assess their genetic response to pathogen infection, we sprayed *Magnaporthe oryzae* conidia suspension with 0.005% Tween on to 3-week-old plants of Dongjin rice and then placed them in containers for 24 h to maintain 80% humidity under darkness before transferring them to a chamber under a 14-h photoperiod. Control and mock samples were collected by spraying water without and with 0.005% Tween, respectively.

### RNA extraction and quantitative PCR

Leaf samples were frozen in liquid nitrogen and homogenized with a TissueLyser II (Qiagen, Hilden, Germany). Total RNA was extracted using RNAiso Plus according to the manufacturer's protocol (Takara Bio, Kyoto, Japan). The qPCR was performed by Qiagen Rotor-Gene Q real-time PCR cycler using follow thermal cycling procedure: 95°C for 10 s, 60°C for 30 s, and 72°C for 1 min. For evaluating tissue-specific expression patterns by real-time PCR, we used *rice ubiquitin 5* (*OsUbi5, LOC_Os01g22490*) (Jain et al., 2006) as an endogenous control to normalize variance in the quality of RNA and the amount of cDNA. *LATE ELONGATED HYPOCOTYL* (*OsLHY*) served as the positive control when examining diurnal rhythms. The effects of cold and heat stress were monitored using *OsNAC6* (*LOC_Os01g66120*) (Ohnishi et al., 2005) and *OsHSP1* (*LOC_Os04g01740*) (Moon et al., 2014), respectively, as positive controls. Successful interaction between rice and *M.oryzae* was convinced by positive control *OsPR10a* (*LOC_Os12g36880*) (Lee et al., 2009). All of the primers for these real-time analyses are shown in Table S1.

### Co-expression analysis

Twenty-five co-expressed genes of 6 individual *MLO* genes (*OsMLO1, 2, 3, 4, 8*, and *9*) with top-score Pearson Correlation Coefficient (PCC) value were collected, based on the co-expression tool installed in Genevestigator (Zimmermann et al., 2004). Co-expressed genes might provide useful functional clues for associated *MLO* genes. Especially, functionally characterized genes collected from The Overview of functionally characterized Genes in Rice online (OGRO) database (Yamamoto et al., 2012) are more effective for this purpose.

## Results

### Integration of anatomical expression patterns with a phylogenetic tree of the rice *MLO* family

**Figure 1:**
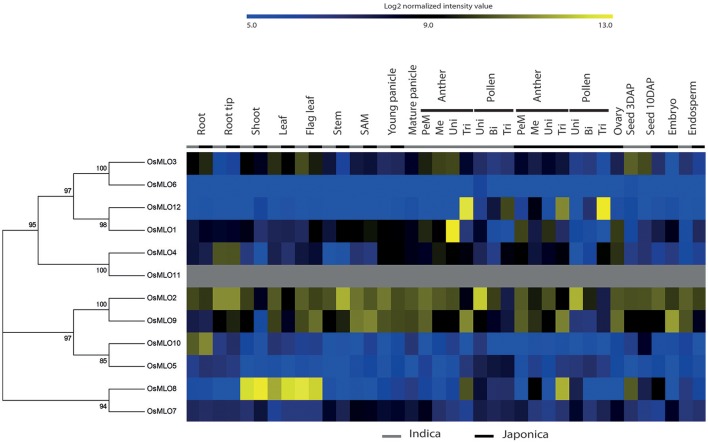
**Meta-analysis of ***OsMLO*** gene expression patterns using 983 Affymetrix anatomical array data**. Blue, low level of log_2_ intensity; yellow, high level. Gray bar, *indica* samples; black bar, *japonica* samples. *OsMLO11* has no probe on rice Affymetrix chip. SAM, shoot apical meristem; PMe, pre-meiotic; Me, meiotic; Uni, uninucleate stage; Bi, binucleate stage; Tri, trinucleate stage; DAP, days after pollination.

Using protein sequences for 12 *MLO* genes in rice, we generated a phylogenetic tree that incorporated subgroup information and anatomical meta-expression profiles based on 983 Affymetrix array data (Cao et al., 2012) (Figure 1). Starting at the top of the tree, *OsMLO3* displayed a medium level of expression in almost all tissues, including roots, shoots, leaves, and flag leaves at the vegetative stage, and anthers and seeds at the reproductive stage. It was most closely linked with *OsMLO6*. However, very low expression by the latter in whole tissues suggested that *OsMLO3* had a more dominant role. Uniquely, *OsMLO*12 showed strong anther-preferred expression in trinucleate and mature pollen. This profile was consistent with that reported by Yi et al. (2014). Expression of *OsMLO1* was high in anthers at the uninucleate stage, especially in *indica* rice, although not at a level similar to that found in uninucleate pollen (Figure S1). Thus, *OsMLO1* might be more active in the anthers than in the pollen grains themselves. The Affymetrix data revealed that *OsMLO4* was preferentially expressed in the root tips but at only a low level in other tissues. In contrast, *OsMLO2* was ubiquitously expressed. One of its paralogs, *OsMLO9*, showed dominant expression patterns in reproductive organs, and transcripts were particularly abundant in anthers at the uninucleate pollen stage while *OsMLO2* was not detected there (Figure S1). Although *OsMLO5* and *OsMLO10*, as well as *OsMLO7* and *OsMLO8*, are thought to have resulted from segmental duplication, each pairing appears to have retained differential expression patterns from an anatomical perspective. While expression of *OsMLO5* was extremely low in the roots, that of *OsMLO10* was much higher than for *OsMLO5*. Moreover, when compared with *OsMLO7, OsMLO8* was more predominantly expressed in the shoots, leaves, and anthers at the trinucleate pollen stage (Figure 1). All of these findings suggested that functional specificity and dominance is much greater for *OsMLO8* and *OsMLO10* than for their homologs *OsMLO7* and *OsMLO5*, respectively. Therefore, with the exception of *OsMLO11*, for which no probe was printed on the Affymetrix chip, we were able to use meta-expression data to estimate the anatomical functions of all *MLO* genes examined here.

### Confirmation of anatomical expression patterns for rice MLO family genes by real-time

**Figure 2:**
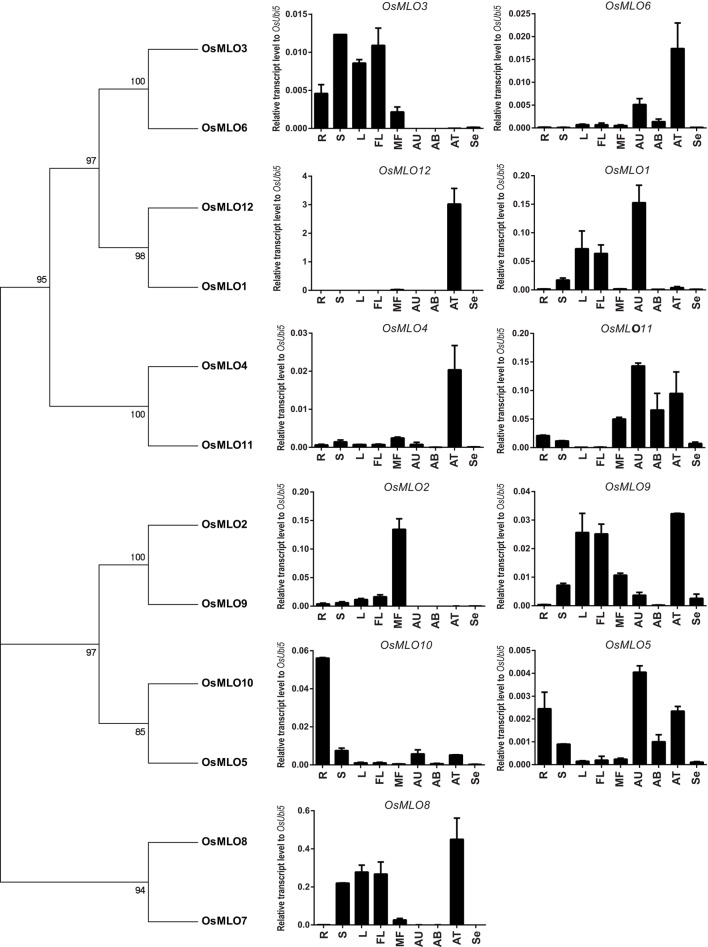
**Real-time expression profiles for 12 ***OsMLO*** genes**. Anatomical samples were prepared from root (R), shoot (S), mature leaf (L), young panicle (3cm-YP), mature flower (MF), anther [uni- (AU), bi- (AB), and tricellular (AT)], and seed at 6 days after pollination (Se). *Rice ubiquitin* (*OsUbi5*) was served as internal control.

To confirm the results from our meta-analysis of anatomical expression profiles, we performed real-time PCR with 12 rice *MLO* genes that are expressed in the roots, shoots, leaves, young panicles, mature flowers, and seeds (at 6 d post-pollination), as well as in anthers sampled at the uni-, bi-, and trinucleate stages (Figure 2). Our findings here closely matched those obtained from meta-analyses of tissue-specific expression profiles. However, we failed to detect any expression of *OsMLO7*, probably because its transcript level was extremely low. We determined that *OsMLO11* was highly expressed in mature flowers and in uni-, bi-, and trinucleate anthers. These results demonstrated the high reliability of meta-expression profiles based on a large collection of transcriptome data.

### Phylogenetic and comparative expression analyses of MLO proteins in rice, barley, and *Arabidopsis*

Although the roles of rice MLO family members have long been studied, only one of those genes has known biological functions. It is known that *OsMLO12* regulates pollen hydration (Yi et al., 2014). However, we believed that the expression patterns of other genes in that family might be good indicators for predicting their biological functions. We also expected that a comparative transcriptome analysis of MLO family members from rice and *Arabidopsis* would provide more accurate evidence for functional conservancy among orthologous gene groups in those two species. Therefore, we combined phylogenetic and meta-expression data from Genevestigator, which uses a similar platform to present expression patterns within an anatomical context.

**Figure 3:**
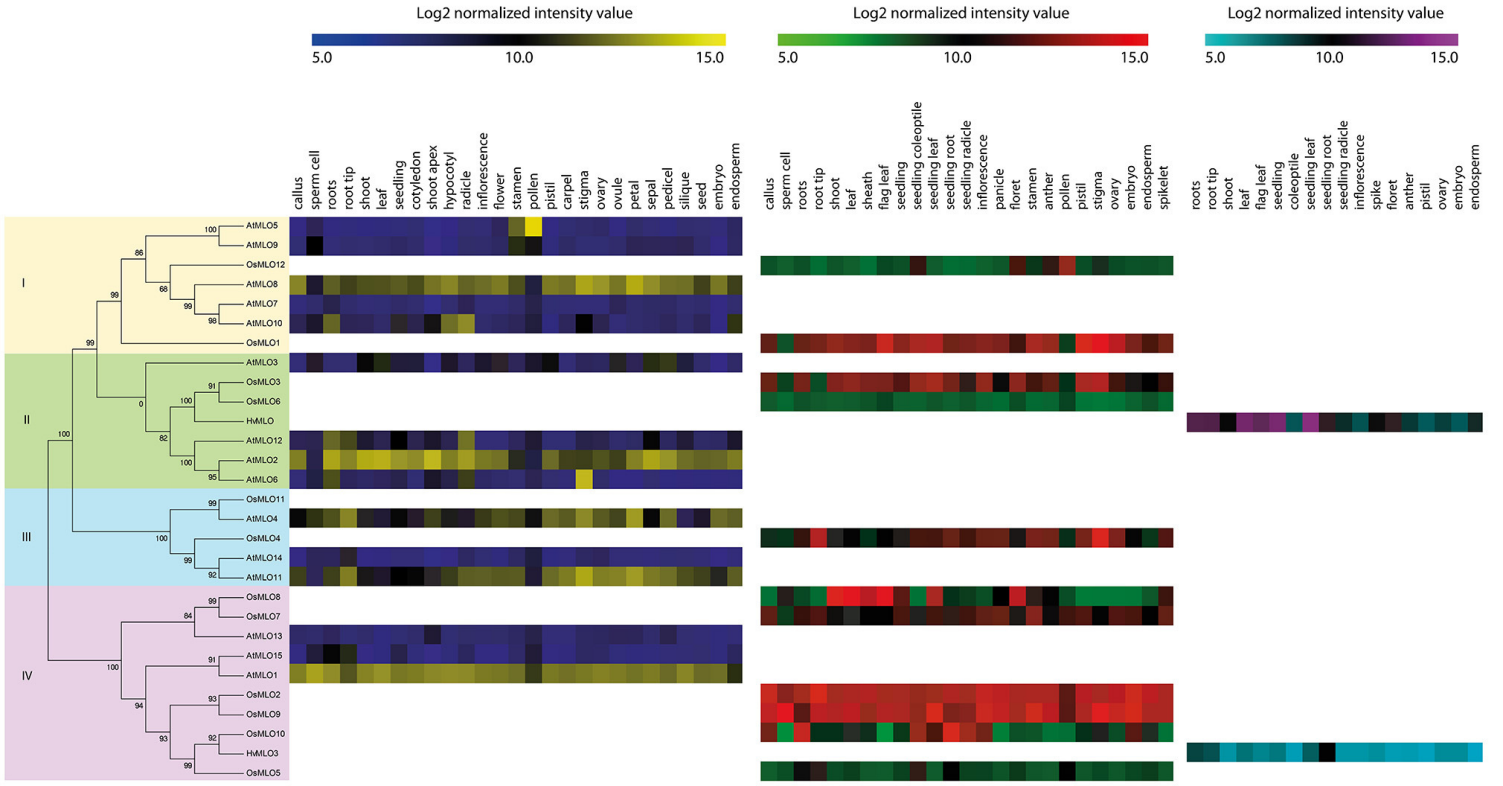
**Comparisons of expression patterns among ***OsMLO*** genes**. Log_2_ intensity values (colored by species) ranged from 5 to 15: *Arabidopsis thaliana* (blue to yellow), *Oryza sativa* (green to red), and *Hordeum vulgare* (cerulean to violet). *OsMLO11* expression pattern was not available in Genevestigator data. Clade number is indicated on left side of phylogenetic tree.

Our phylogenetic tree covered 29 protein sequences that were aligned from rice (12 MLOs), *Arabidopsis* (15), and barley (2). This established four clades, i.e., I through IV (Figure 3). Dividing the number of clade for MLO family has yet to be unanimous (Feechan et al., 2008; Pessina et al., 2014; Kusch et al., 2016), we separated 29 MLOs into four clades base on primary evolution and relative known function gene with small numbers of *MLO* genes. This tree implied that the evolutionary processes for *MLO* genes from monocots and dicots were rather independent. For example, the 29 members included only two orthologous pairings—*AtMLO4*/*OsMLO11* in Clade III and *OsMLO10*/*HvMLO3* in Clade IV.

For Clade I, the anatomy tool installed in Genevestigator indicated similar expression between *AtMLO5* and *OsMLO12* (Figure 2). Both *AtMLO8* and *OsMLO1* were ubiquitously expressed except in the pollen (Figure 2). *AtMLO10* was predicted to function in the roots, hypocotyls, radicles, and endosperm.

In Clade II, expression of *OsMLO3* in almost all organs/tissues was the most similar to the patterns found for *AtMLO2* than *AtMLO6*, and *AtMLO12* expression. Elliott et al. (2002) have demonstrated that introducing *OsMLO3* into the barley MLO null mutant genotype *mlo-5* leads to 50% susceptibility restoration. Therefore, it appears that *OsMLO3* in rice might have a biological function in plant defenses. However, it is unclear whether the presence of a single copy of that gene is sufficient to confer pathogen resistance. Because *OsMLO6* was expressed at low levels in most tissues/organs while *AtMLO6* and *AtMLO12* were highly expressed in roots and radicles, we suspect that the latter two genes have roles in root development that are conserved with *AtMLO2*.

For Clade III, the expression patterns of *AtMLO4, AtMLO11*, and *OsMLO4* indicated that they have similar functions in the development of root tips and stigma, suggesting that the roles of *MLO* genes within that clade are conserved. Although data for *OsMLO11* were not available using the Affymetrix array platform, we were able to determine its spatio-temporal expression patterns using the Agilent array platform. In doing so, we found that this gene was highly expressed in the roots, inflorescences, pistils, palea/lemma, and ovaries (Figure S2). Therefore, expression was similar between *OsMLO11* and *OsMLO4* at the reproductive stage. While *AtMLO4* displayed similar expression to *AtMLO11* in every tissues/organ, *AtMLO14* expression is disparity. However, whereas knockouts of *AtMLO4* and *AtMLO11* caused an abnormal root phenotype, the paralog *AtMLO14* was not involved in root development. This might explain their different patterns of expression even though *AtMLO11* and *AtMLO14* were the closest members in the evolutionary tree (Figure 3). Nevertheless, we expect that the *osmlo4* mutant would display the same behavior upon tactile stimulation as that shown by mutants of *AtMLO4* or *AtMLO11*.

In Clade IV, *OsMLO8* was preferentially expressed in the leaves and shoots. However, we were unable to find similar expression patterns for *Arabidopsis* genes in this clade. Instead, *AtMLO1, OsMLO2*, and *OsMLO9* were ubiquitously expressed in most of the anatomical samples, suggesting that these MLO family members have housekeeping functions.

### Diurnal regulation associated with the MLO family

**Figure 4:**
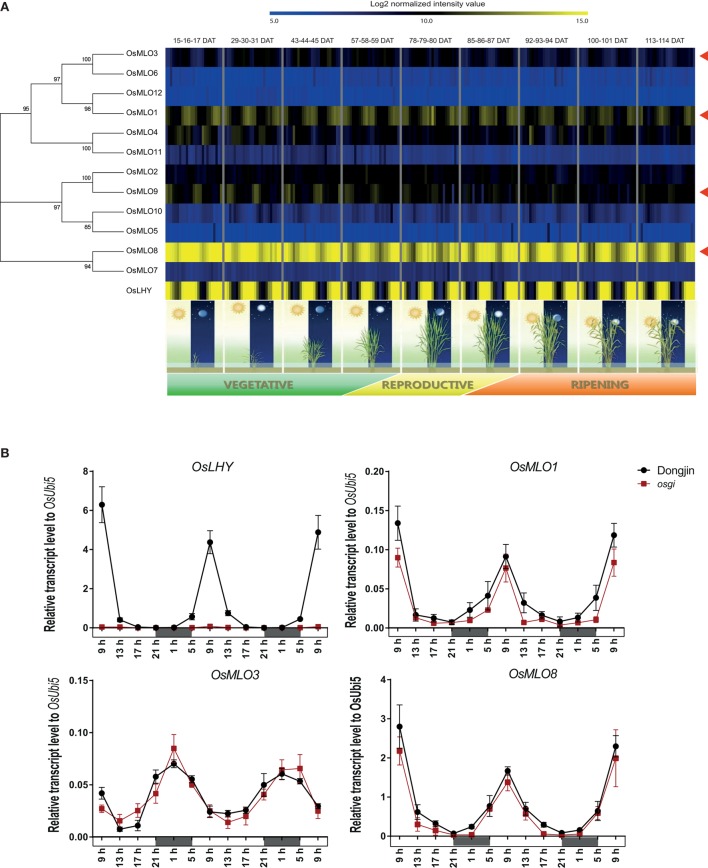
**Diurnal expression patterns of ***OsMLO*** genes (red arrows indicate diurnal rhythm) in mature leaves, using available Agilent 44k array data over entire plant life span (A), or evaluated at 13 time points over 48-h period in “Dongjin” rice and ***osgi*** mutant (B)**. As a standard marker gene for diurnal rhythm, expression of *OsLHY* was peaked during daytime. *OsUbi5* was served as internal control. DAT, days after transplanting. Continuous white and black bar indicates day and night time, respectively.

Diurnal rhythm is synchronized with the day/night cycle and is regulated by two mechanisms: light and the circadian clock. To elucidate the diurnal rhythm of our *MLO* genes, we analyzed their expression patterns using publicly available Agilent 44k array data obtained for diurnal and circadian gene expression in the leaf over the entire life span of field-grown rice plants (Sato et al., 2013). Among the 12 *MLO* genes, leaf preferentially expressed *OsMLO8* showed high expression that was obviously diurnal, while *OsMLO1, OsMLO3*, and *OsMLO9* were also diurnally expressed, albeit at a more moderate level, in mature leaves (Figure 4A). Real-time PCR analyses of genes from 4-week-old leaves indicated that *OsMLO1, OsMLO3*, and *OsMLO8* exhibited cyclic expression every 24 h (Figure 4B).

To obtain insight into the mechanism that regulates the diurnal rhythm of *MLO* genes, we evaluated a rice mutant. In *osgi*, diurnal expression of marker gene *OsLHY* was dramatically down-regulated across all time points whereas the rhythm of expression for *OsMLO1, OsMLO3*, and *OsMLO8* was similar to that detected in WT plants (Figure 4B). These findings indicated that the genes are not related to *OsGI* or the circadian mechanism.

### Light response associated with the MLO family

**Figure 5:**
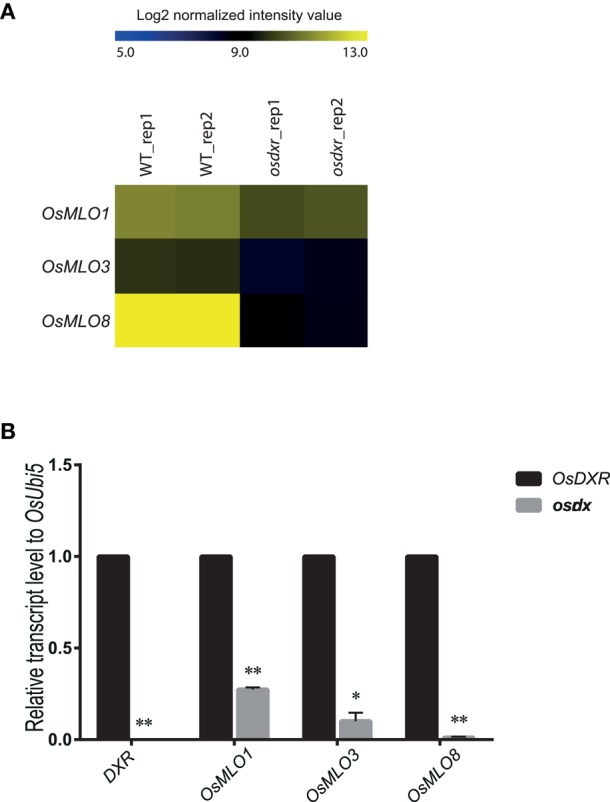
**Heatmap for light- or dark-inducible expression of ***OsMLO*** genes in wild-type (WT/DXR) rice and ***osdxr*** mutant**. **(A)** Analysis of 3 genes using Agilent 60K microarray data for *osdxr* mutant. **(B)** Validation of expression of *OsMLO1, OsMLO3*, and *OsMLO8* in WT (DXR) and *osdxr* mutant. Transcripts of *OsDxr* was absent in knockout plant. ^**^p < 0.01; ^*^0.01 < *p* < 0.05.

To determine whether the *MLO* genes are involved in light-/dark-response mechanisms, we evaluated their expression using Agilent 60K microarray data for a mutant of the light-responsive *osdxr*, which belongs to the methylerythritol 4-phosphate pathway (Jung et al., 2008) (Figure 5A). Real-time PCR analysis confirmed that *OsMLO1* expression was decreased while that of *OsMLO3* was much lower in the mutant than in the WT (Figure 5B). Leaf-specific expression of *OsMLO8* was suppressed completely in *osdxr*, suggesting a close connection between *OsMLO8* and the light-response pathway. Our results implied that the *MLO* genes are diurnally expressed and that *OsMLO3* is dark-inducible while *OsMLO1* and *OsMLO8* are light-inducible (Figure 5).

**Figure 6:**
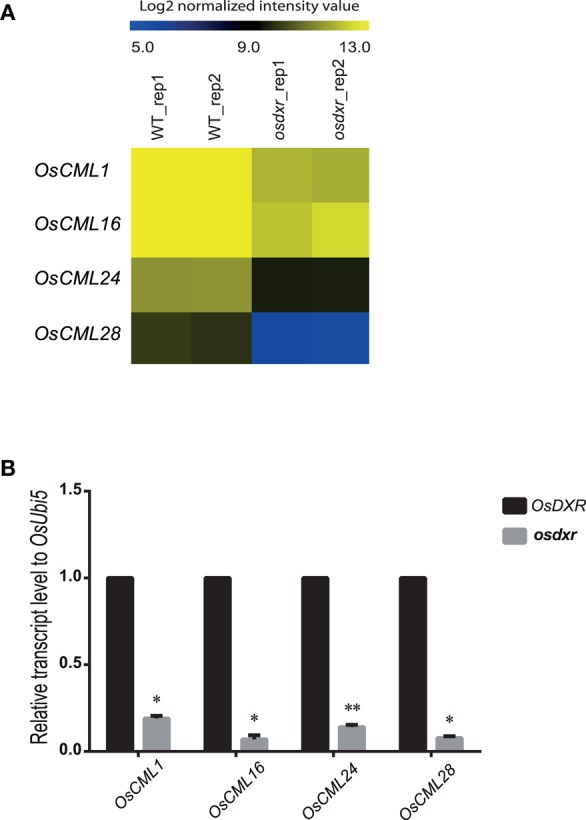
**Heatmap for expression patterns of ***OsCML*** genes in wild-type (WT/DXR) rice and ***osdxr*** mutant. (A)** Expression analysis of 4 *OsCML* genes using Agilent 60K microarray data for WT vs. *osdxr* knockout line. **(B)** Downregulation of *OsCML* genes in *osdxr* mutant demonstrated by real-time PCR analysis. ^**^p < 0.01; ^*^0.01 < *p* < 0.05.

The MLO proteins interact with calmodulin as the second messenger to transfer a signal forward to downstream pathways (Kim et al., 2002a,b; Stein and Somerville, 2002). To investigate whether the diurnal response-dependent *OsMLO*s function with calmodulin proteins, we examined the differential expression of *calmodulin* (CaM) and *CaM-like* (CML), searching for genes with log_2_-fold changes >1.8 and *p*-values <0.05 in the *osdxr* mutant (Table S2). Subsequent analysis of the microarray data presented four *CML* genes—*OsCML1, OsCML16, OsCML24*, and *OsCML28*—with decreased expression in that mutant (Figure 6A). Real-time PCR analysis also confirmed that all four were significantly down-regulated in *osdxr* (Figure 6B). Therefore, we propose that these OsCML genes would be primary targets for studying functional relationships among diurnal response-dependent OsMLOs.

### Responses of the *MLO* family to high- and low-temperature stresses

**Figure 7:**
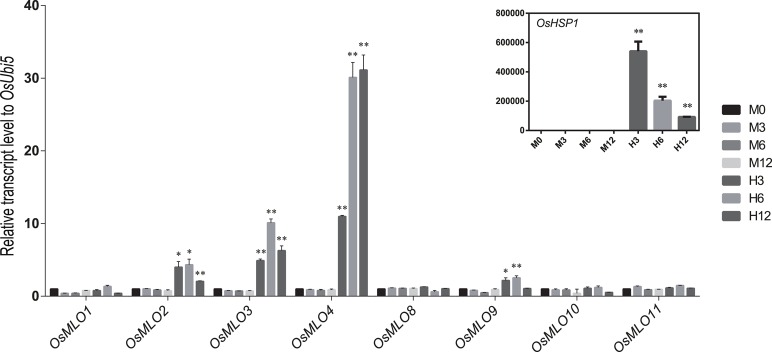
**Expression profiles of ***OsMLO*** genes under heat stress evaluated by real-time PCR analysis**. *OsHsp1* served as a positive marker for heat-stress response. *OsUbi5* was used as internal control. M, control treatment; H, high-temperature treatment. Numbers after M and H indicate time points (hours) after stress treatment. ^**^p < 0.01; ^*^0.01 < *p* < 0.05.

Although the barley *MLO* genes are affected by abiotic stresses such as leaf-wounding and herbicides (Piffanelli et al., 2002), little is known about how genes in that family are influenced by other sources of stress. We used the log_2_ fold-change data in response to abiotic stresses (Figure S3) to investigate the meta-expression patterns of 12 rice *MLO* genes. Differential expression was monitored via real-time PCR with samples under either heat- or cold-stress conditions. As expected, *OsMLO2, OsMLO3, OsMLO4*, and *OsMLO9* were rapidly up-regulated during the first 3 h of heat treatment (Figure 7). Their expression began to decline after 12 h of treatment but was still higher than that measured from the WT control. Among those four genes, *OsMLO4* was the most responsive to high temperature, with expression being >30-fold higher than that in control plants after 6 h and remaining at that level for 12 h of treatment. Because *OsMLO5, OsMLO6*, and *OsMLO7* were expressed only at low levels in the leaves, they were eliminated from further analysis of temperature sensitivity.

**Figure 8:**
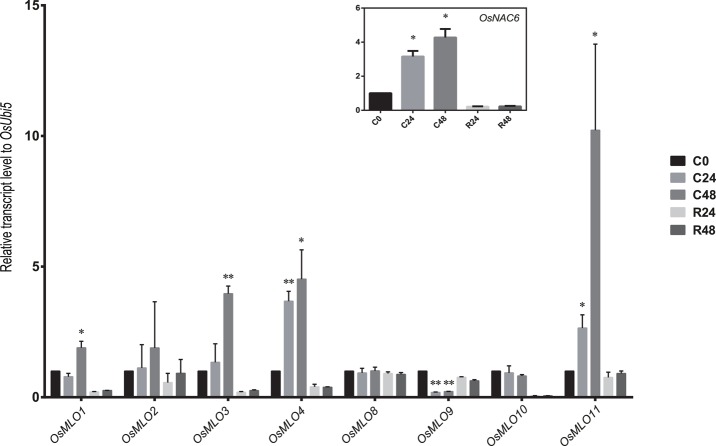
**Expression profiles of ***OsMLO*** genes under cold stress evaluated by real-time PCR**. *OsNAC6* served as a positive marker for cold-stress response. *OsUbi5* was used as internal control. C, cold treatment; R, recovery. Numbers after C and R indicate time points (hours) after stress treatment. ^**^p < 0.01; ^*^0.01 < *p* < 0.05.

In response to cold stress, *OsMLO1* and *OsMLO3* were up-regulated at 48 h after treatment was applied. However, their level of expression declined to that measured from the control when the chilled plants were allowed to recover at 28°C (Figure 8). Both *OsMLO4* and *OsMLO11* were quickly induced by low temperatures, with expression increasing after just 24 h of treatment and transcripts being retained at higher levels until Hour 48. By contrast, *OsMLO9* was down-regulated by 24 h of chilling. This response was the opposite of its upregulation by heat stress, implying that the gene has separate functions in determining the plant response to temperature extremes. All of these findings provided evidence that the expression of these rice *MLO* genes is influenced by cold and/or heat stress.

### Interactions between rice MLO genes and *Magnaporthe oryzae*

**Figure 9:**
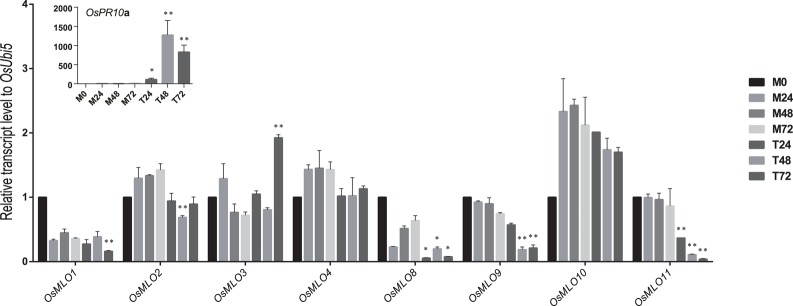
**Real-time analysis of ***OsMLO*** genes in Dongjin rice variety inoculated with ***M. oryzae*** PO6-6**. Samples from mock treatment (M), and fungal inoculation treatment (T) were examined after inoculation for 24, 48, and 72 h. Numbers after C and R indicate time points (hours) after stress or MOCK treatment. Rice *PR10a* served as a positive marker for infection. *OsUbi5* was used as internal control. ^**^p < 0.01; ^*^0.01 < *p* < 0.05.

“Dongjin” rice plants were inoculated with *M. oryzae* PO6-6 to monitor the response of *MLO* genes to pathogen infection. We had expected that *OsMLO3* would show differential expression similar to that demonstrated by its homologous members in *Arabidopsis* and barley. In fact, *OsMLO3* was significantly induced at 72 h after infection (Figure 9). Expression of two light-inducible genes—*OsMLO1* and *OsMLO8*—was relatively decreased after 72 h and 24 h, respectively, two closely linked members—*OsMLO2* and *OsMLO9*—were slightly down-regulated at 48 h, and *OsMLO11* were significantly reduced expression at 48 h after treatment. These data indicated that rice MLO family members have possible roles in the pathogen response.

### Co-expression analysis suggests potential downstream genes of MLO signaling

Twenty five co-expressed genes of 6 individual *MLO* genes (*OsMLO1, 2, 3, 4, 8*, and *9*) with the highest PCC value were selected by co-expression analysis tool installed in Genvestigator with 2532 anatomical samples (Table S3). To identify the possible function in which each *MLO* gene is involved, we searched rice genes with known functions from the OGRO database out of the co-expressed genes. Unfortunately, progress of functional genomics in rice is still restricted and explains functions for 2 or 3% of genes in rice genome (Chandran et al., 2016). Thus, we only found two known genes from co-expressed genes to *OsMLO3* or *OsMLO8*. A known gene, *OsHsfA4d*/*Spl7*, one of co-expressed genes to *OsMLO3*, encodes heat shock transcription factor (Yamanouchi et al., 2002), which activates heat shock genes under high temperature stress. *spl7* mutant causes scattered brown spot on leaf and dead cells increase since exposing under heat stress or UV solar radiation. *spl7* also accumulated higher amounts of H_2_O_2_ in response to rice blast fungal elicitor (Kojo et al., 2006). Although the co-expression score (PCC value) of *OsMLO3* and *Spl7* is 0.6847, this linkage suggests one of downstream responsive genes of *OsMLO3* associated with heat stress, cell death and H_2_O_2_ accumulation. In case of *fructose-1,6-bisphosphatase* (*OscFBP1*/*MOC2*), the co-expression score with *OsMLO8* is 0.9314. *OscFBP1* converts triose-phosphates to sucrose in the day (Daie, 1993). Loss function of *OscFBP1* exhibits monoculm phenotype and reduced photosynthetic rate (Lee et al., 2008; Koumoto et al., 2013). Since leaf- preferred *OsMLO8* gene showed the peak of expression level under daylight, its co-expression with *OscFBP1* suggests the important role in photosynthesis.

## Discussion

Although members of the MLO family generally function in defense responses, some of those genes are also involved in developmental processes, such as pollen tube elongation or root formation (Chen et al., 2009; Kessler et al., 2010; Bidzinski et al., 2014). The functional implication of each MLO member can be ascertained by meta-expression data based on a large collection of transcriptome data. Expression patterns for genes associated with the development of pollen tubes or roots are closely linked with their corresponding tissues/organs, thereby making them useful indicators when examining currently uncharacterized *MLO* genes. Phylogenomic analysis combined to whole-genome transcriptome provides diverse information about the function of *MLO*s. The further studies can be followed to address the biological processes of each *MLO* gene by using protein-protein interaction or co-expression analysis (Jin et al., 2013; Nguyen V. N. T. et al., 2014). In particular, their patterns of expression in response to cold or heat stress can give researchers novel directions for functional studies of this gene family. Those tools also allow us to estimate functional redundancy among closely linked family members and can inspire more effective strategies for identifying defective phenotypes in loss-of-function studies. For example, although *AtMLO2, AtMLO6*, and *AtMLO12* are redundant, their roles are not equal in conferring a defense response against fungi. Likewise, single knockout mutants of *AtMLO4* and *AtMLO11* show a similar root-wave phenotype, but this phenotype is not enhanced in a double mutant. Among *MLO* genes in rice, *OsMLO3* and *OsMLO6* are supposed to be originally tandem duplication (Liu and Zhu, 2008). We also determined that *OsMLO3* and *OsMLO6* are closely related from our phylogenomic data, but expression of *OsMLO3* is stronger than that of *OsMLO6*, meaning that *OsMLO3* might have a predominant role between them. Additionally, two pairs of *OsMLO10*/*OsMLO5* and *OsMLO8*/*OsMLO7* are believed to be the result of segmental duplication, but the stronger and unique level of expression of *OsMLO10* and *OsMLO8* suggests their predominant roles relative to their closest members. These results suggest that functional redundancy in rice MLO family might occur to maintain robustness of few members which might play the important roles in rice during evolution.

One advantage of a phylogenomic analysis is that it informs researchers about which experimental conditions are most suitable when facilitating studies of genes of interest to identify defective phenotypes. Here, we identified MLO genes associated with specific tissue/organ types, including *OsMLO12*, which is uniquely expressed in pollen at the trinucleate stage (Yi et al., 2014); *OsMLO10*, which is preferentially expressed in the roots; and *OsMLO8*, which is preferentially expressed in shoots and in anthers at the trinucleate stage. Because ubiquitous expression of the MLO family members may not be beneficial at all stages of plant development, it is critical that we make careful selections of those genes that have conditional or tissue-preferential regulation when we are developing more desirable rice cultivars. Our combination of microarray data and real-time results demonstrated that *OsMLO1, OsMLO2, OsMLO3, OsMLO4, OsMLO9*, and *OsMLO11* are involved in the response to heat and/or chilling in rice. This suggests that MLO proteins have roles in plant adaptations to temperature stresses. Under those stressful conditions, accumulations of reactive oxygen species (ROS) act as a signal for plant defense response (Knight and Knight, 2001), even that phenomenon can put plants at risk. Hence, exposure to extreme temperatures is a stress that quickly induces H_2_O_2_ accumulations and damages plants (O'Kane et al., 1996; Rizhsky et al., 2002; Vacca et al., 2004). Besides, other researchers have reported a negative relationship between MLO protein activity and H_2_O_2_ accumulations under biotic stress (Opalski et al., 2005; Kim and Hwang, 2012). Thus, we suggest that the absence of MLO proteins in rice might cause a reduction of H_2_O_2_ that enhances tolerance against those environmental challenges. Nevertheless, the biochemical functions of MLO proteins remain to be elusive.

Even though the functions of MLO proteins may have diverged evolutionarily between species, the rice MLO family appears to have a role in defenses against fungal attacks. Piffanelli et al. (2002) initially showed that the induction of hydrogen peroxide (H_2_O_2_) accumulations upon infection by a fungal pathogen was stronger in a barley *mlo* mutant than in the WT. And in this study, upregulation of *OsMLO3* at 72 h after pathogen infection might induce the production and accumulation of H_2_O_2,_ leading to cell death in rice leaves. However, it is unclear why *OsMLO1, OsMLO2, OsMLO8, OsMLO9*, and *OsMLO11* are down-regulated by such infections.

The role of *MLO* genes has never been mentioned as light responsive genes. In this study, we identified that *OsMLO1, OsMLO3*, and *OsMLO8* in particular showed diurnal behavior during the light/dark cycle. This result suggests that changing day/night cycle effects on the expression of those *MLO* genes.

Altogether, *MLO* genes in rice are supposed to share their roles into different processes of development and defense. The seven transmembrane motif-containing MLO proteins perhaps play the role as the perception of various stimuli from environment. Since plants are exposed to variety of stress, increasing intracellular Ca^2+^ level results in change the level of H_2_O_2_ through binding to CaM/CML, ubiquitous calcium-binding protein (McCormack et al., 2005; Reddy et al., 2011; Lee et al., 2012). Moreover, CaM was discovered to be important for defense response in plant by the interaction with MLO (Kim et al., 2002b). This finding suggested the potential communication between plant abiotic responses and immunity via signaling from MLO to Ca^2+^—dependent CaM/CML, which stimulates the production of H_2_O_2_. Additional, co-expression gene of *OsMLO3* also convinced the relationship between heat stress and cell death since *spl7* mutant increased death cell under heat stress and retained more H_2_O_2_ than wildtype. Although no direct evidence of an association between the light pathway and ROS signaling has been described previously, photoperiod is critically related to oxidative signaling. In *Arabidopsis*, the *catalase2* mutant causes oxidative signaling to be activated during short days but not long days, specifically under photorespiratory conditions (Queval et al., 2007). A link between day length and H_2_O_2_ accumulations has also been demonstrated with transcriptome data showing that many H_2_O_2_-responsive genes are day length-dependent (Queval et al., 2012). As we have suggested here, a MLO mediating relationship among abiotic stress, biotic stress, photoperiod, and H_2_O_2_ might exist in rice.

**Figure 10:**
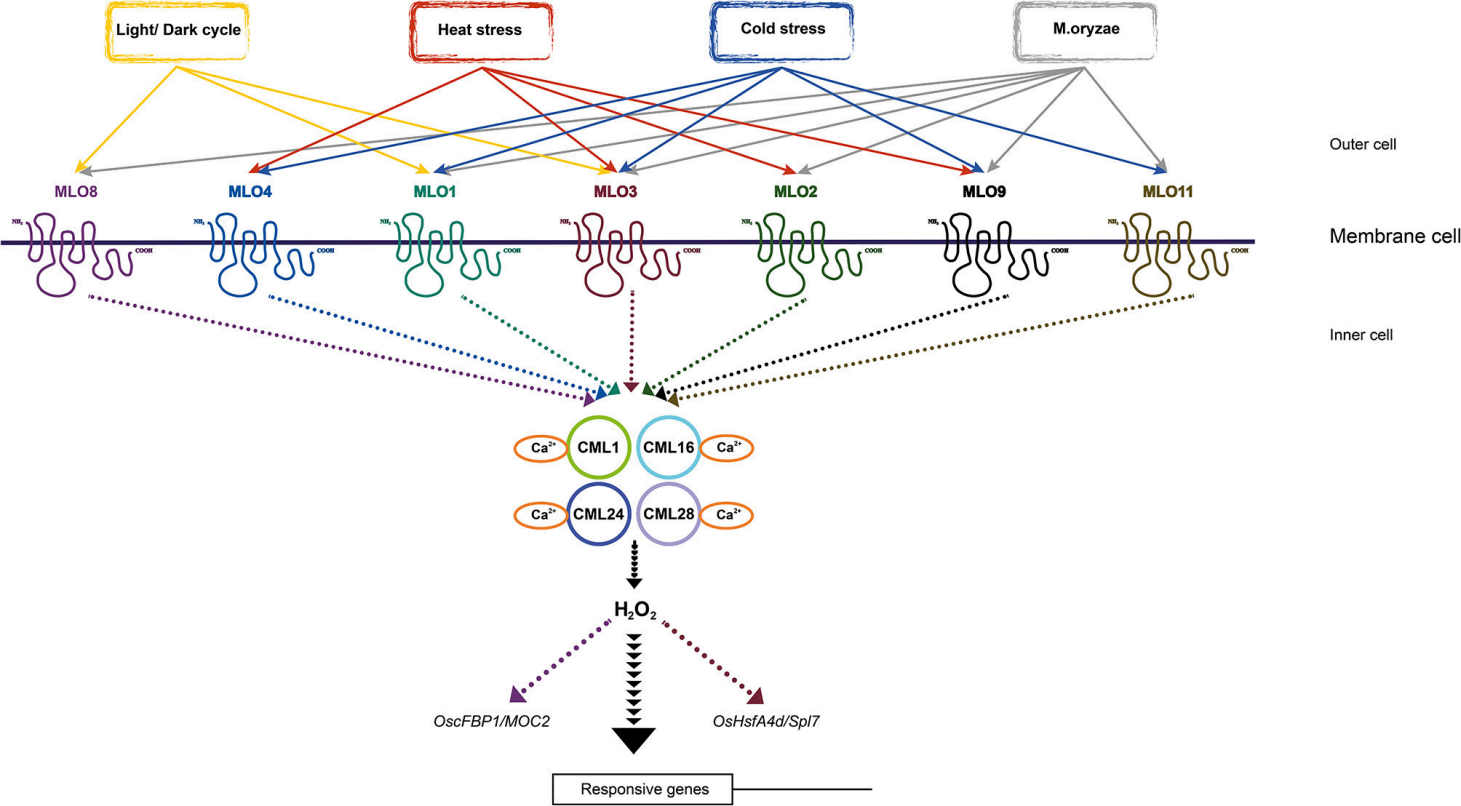
**Simplified model of OsMLO mediating stress-response pathways**. Model includes 7 *OsMLO* genes that respond to different experimental conditions, plus *OsCML* genes in downstream signaling pathway of *OsMLO* family genes and potential responsive genes in signaling pathways. *OsHsfA4d*/*Spl7* and *OscFBP1*/*MOC2* were presented as downstream factors of *OsMLO3* and *OsMLO8*, respectively. Solid arrows show relationships analyzed in this study; dashed arrows indicate unclear relationships. Individual MLO pathway was indicated by different color.

In summary, we have gathered evidence that the MLO family in rice is involved in a light signaling pathway associated with the diurnal rhythm. The results from this work also underscore the roles that these genes have in both abiotic- and biotic-stress responses, as shown in Table 1 and the model depicted in Figure 10. The featured expression patterns were also summarized in Table 1. Environment aspects might stimulate the changing of H_2_O_2_ level by means of the interaction MLO and CaM/CML-Ca^2+^. The production of H_2_O_2_ might play as a messenger and stimulate the expression of the responsive genes to adapt to the environmental change. However, further researches will be required to obtain a deeper understanding of the rice *MLO* genes and their functional implications.

**Table 1:** **Summary of MLO family members in rice with highlighted meta-expression patterns**.


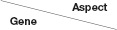	**Preferred tissue(s) revealed by qRT-PCR**	**Diurnal rhythm**	**Heat stress**	**Cold stress**	***Magnaporthe oryzae* infection**
*OsMLO1*	Leaf, uninucleate anther	Light-inducible		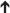 ^a^	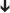 ^b^
*OsMLO2*	Mature flower		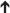 ^a^		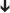 ^b^
*OsMLO3*	Shoot, leaf	Dark-inducible	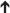 ^a^	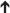 ^a^	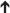 ^a^
*OsMLO4*	Trinucleate anther		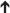 ^a^	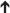 ^a^	
*OsMLO5*	Root, uninucleate anther				
*OsMLO6*	Trinucleate anther				
*OsMLO7*	–				
*OsMLO8*	Leaf, trinucleate anther	Light-inducible			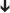 ^b^
*OsMLO9*	Leaf, trinucleate anther		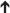 ^a^	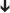 ^b^	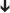 ^b^
*OsMLO10*	Root				
*OsMLO11*	Uninucleate anther			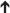 ^a^	
*OsMLO12*	Trinucleate anther				

a *Up-regulated expression relative to untreated control*.

b *Down-regulated expression relative to untreated control*.

## Author contributions

VN and KV performed the experiments; VN, KV, and KJ analyzed the data; and VN, KV, HP, JJ, and KJ wrote the paper. All authors read and approved the final manuscript.

### Conflict of interest statement

The authors declare that the research was conducted in the absence of any commercial or financial relationships that could be construed as a potential conflict of interest.

## Supplementary material

Figure S1 **Details of microarray data for *OsMLO* genes from anthers and pollen of *indica* and *japonica* rice cultivars**. ACP, archespore; PMe, pre-meiotic; Me, meiotic; U, uninucleate stage; B, binucleate stage; T, trinucleate stage; FL, anther at flowering time; MP, mature pollen; GP, geminated pollen.

Figure S2 **Agilent 44K data of *OsMLO* genes, including expression patterns in 13 tissues/organs**. Log_2_ intensities range from 5 (blue) to 15 (yellow).

Figure S3 **Expression patterns based on Affymetrix arrays**. Phylogenomic analysis of 12 *OsMLO* genes with integration of abiotic stress meta-data obtained in response to drought, salt, cold, heat, or submergence. Red boxes, genes up-regulated by stress treatment relative to control; green boxes, down-regulated by stress treatment; gray boxes, data not considered in microarray analysis.

Table S1 **Primer sequences used for real-time/RT PCR and genotyping analyses**.

Table S2 **Details of microarray data for *OsCaM* and *OsCML* genes examined in this study with wild-type rice and *osdxr* mutant**.

Table S3 **Summary of co-expression genes with *OsMLO1, 2, 3, 4, 8*, and *9* genes in 2532 anatomical samples using co-expression analysis tool in Genevestigator**.
